# Neuro-Ophthalmological and Musculoskeletal Complications Due to Disseminated Klebsiella pneumoniae Infection: A Case Report and Proposed Management Framework

**DOI:** 10.7759/cureus.104569

**Published:** 2026-03-02

**Authors:** Grace Fong

**Affiliations:** 1 Internal Medicine, Sengkang General Hospital, Singapore, SGP

**Keywords:** bacterial liver abscess, klebsiella pneumoniae (kp), midbrain abscess, parinaud syndrome, pyogenic osteomyelitis, third cranial nerve (oculomotor nerve) palsy, weber syndrome

## Abstract

*Klebsiella pneumoniae *is known to cause pneumonia, urinary tract infections, bacteremia, meningitis, and liver abscesses in immunocompromised individuals. A distinct invasive syndrome has been reported in Southeast Asia in the past two decades, whereby *K. pneumoniae* results in liver abscesses and extrahepatic complications in immunocompetent individuals. We present a case of disseminated *K. pneumoniae *infection, with metastatic infections of midbrain abscess, liver abscess, and osteomyelitis. A 48-year-old female with a history of chronic alcohol intake, hypertension, and gout presented to our emergency department with diplopia and right ptosis. A right third nerve palsy was noted on admission, and she subsequently developed right fourth nerve palsy, left hemiplegia (Weber's syndrome), and left partial Parinaud syndrome over the course of her hospitalization. This case illustrates both common and uncommon complications of invasive *K. pneumoniae* infection. We also propose a framework for the diagnosis and management of brain abscess.

## Introduction

*Klebsiella pneumoniae* is a Gram-negative, encapsulated, rod-shaped bacterium found in gut and oral flora. It is known to cause pneumonia, urinary tract infections, bacteremia, meningitis, and liver abscesses in immunocompromised individuals [1]. However, in the past two decades, a distinct invasive syndrome has been reported in Southeast Asia, whereby liver abscesses and extrahepatic complications (e.g., meningitis, brain abscess, necrotizing fasciitis, endophthalmitis) are described in immunocompetent individuals infected with *K. pneumoniae* [2,3].

We report a case of disseminated infection caused by *K. pneumoniae*, with metastatic infections of the midbrain, liver abscess, and osteomyelitis, presenting with third nerve palsy and subsequent contralateral hemiplegia (Weber's syndrome), fourth nerve palsy, and partial Parinaud syndrome. We also propose a framework for the diagnosis and management of brain abscess.

## Case presentation

A 48-year-old Chinese female with a history of chronic alcohol intake, hypertension, and gout presented to a hospital in Singapore with sudden-onset diplopia associated with right ptosis. This was preceded by a two-week history of right thigh swelling and pain. She did not report pain or headache associated with diplopia. On presentation, her vital signs were as follows: body temperature, 38 degrees Celsius; blood pressure of 108/64 mmHg; heart rate of 117 beats per minute; respiratory rate of 16 breaths per minute; and oxygen saturation of 95% on ambient air. On physical examination, she was alert and oriented with a Glasgow Coma Scale of 15. Cranial nerve examination revealed right ptosis with a large unreactive pupil and paralysis of adduction, elevation, and depression. The right eye was noted to be in the “down and out position”. This was consistent with a right third nerve palsy. Her right anterior thigh was tender on palpation without overlying skin changes. No other abnormal findings were noted.

Blood cultures were obtained and she was started on intravenous co-amoxiclav for empirical cover of unspecified community-acquired sepsis. Initial laboratory investigations are shown in Table 1. Chest and right femur radiography were unremarkable.

**Table 1:** Results of initial laboratory investigations CRP: C-reactive protein, ALP: alkaline phosphatase, GGT: gamma-glutamyl transpeptidase, Ag: antigen, Ab: antibody

Laboratory parameters	Results	Normal range
Haemoglobin	9.2 g/dL	12.0 – 16.0 g/dL
White blood cells	20.20 × 10⁹/L	4.00-10.00 × 10⁹/L
Platelets	714 × 10⁹/L	140-440×10⁹/L
CRP	65.6 mg/L	≤ 4.9 mg/L
Alanine transaminase (ALT)	24 U/L	≤​​​​​​​ 35 U/L
Aspartate transaminase (AST)	26 U/L	≤​​​​​​​ 35 U/L
ALP	409 U/L	35 – 104 U/L
GGT	521 U/L	6 – 42 U/L
Bilirubin, total	12 umol/L	≤​​​​​​​ 21 umol/L
Albumin	32 g/L	35 – 50 g/L
Urea	2.2 mmol/L	2.5 – 7.8 mmol/L
Creatinine	59 umol/L	45 – 84 umol/L
HbA1c	5.6%	≤​​​​​​​ 6.4%
Human immunodeficiency virus (HIV) Ag/Ab Screen	Non-reactive	-

**Figure 1:**
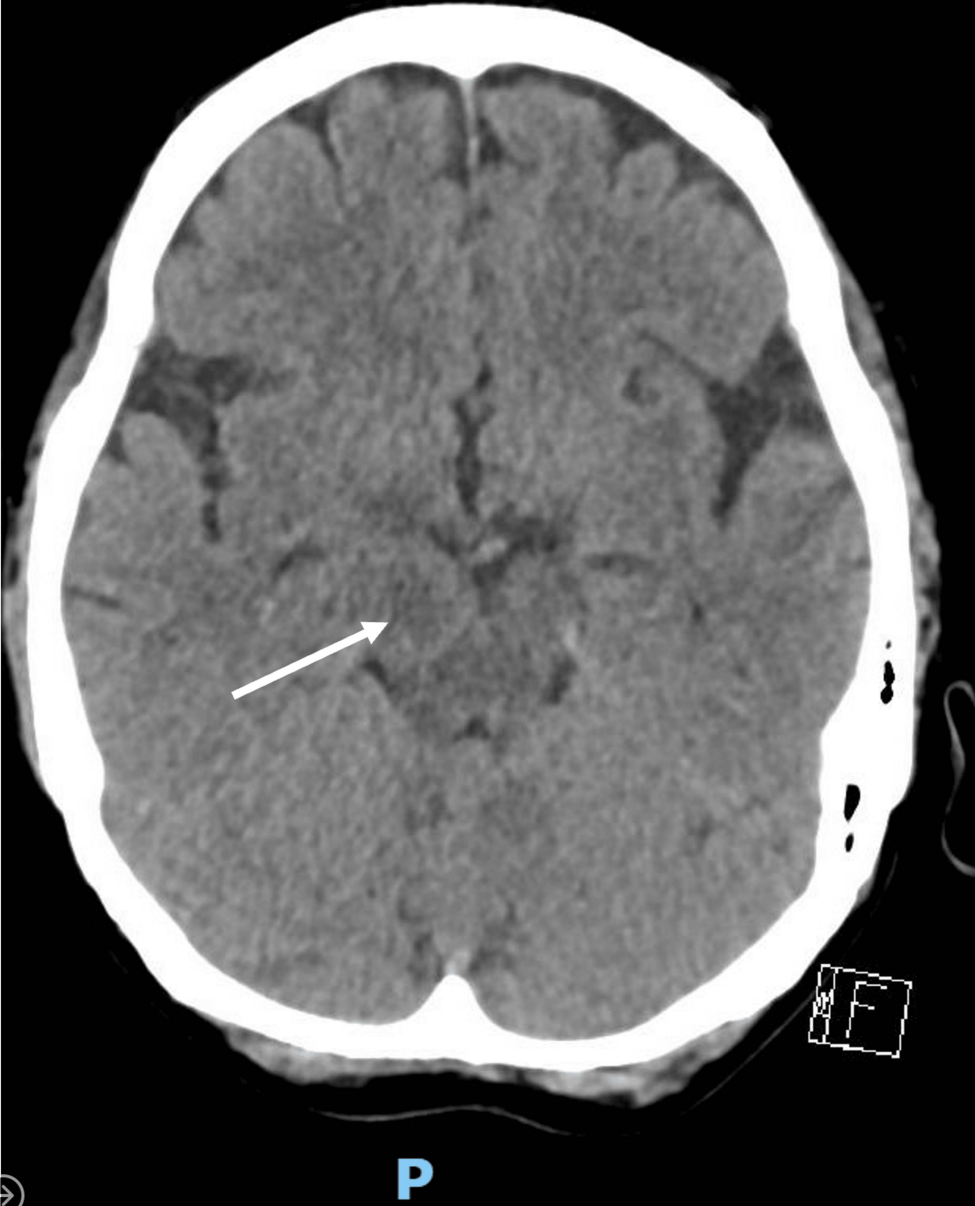
Non-contrast enhanced CT scan of the brain performed on admission demonstrating right cerebral peduncle hypodense lesion (arrow)

Computed tomography (CT) of the brain performed on admission showed a hypodensity measuring 1.6 cm x 1.0 cm within the right cerebral peduncle (Figure 1). 

**Figure 2:**
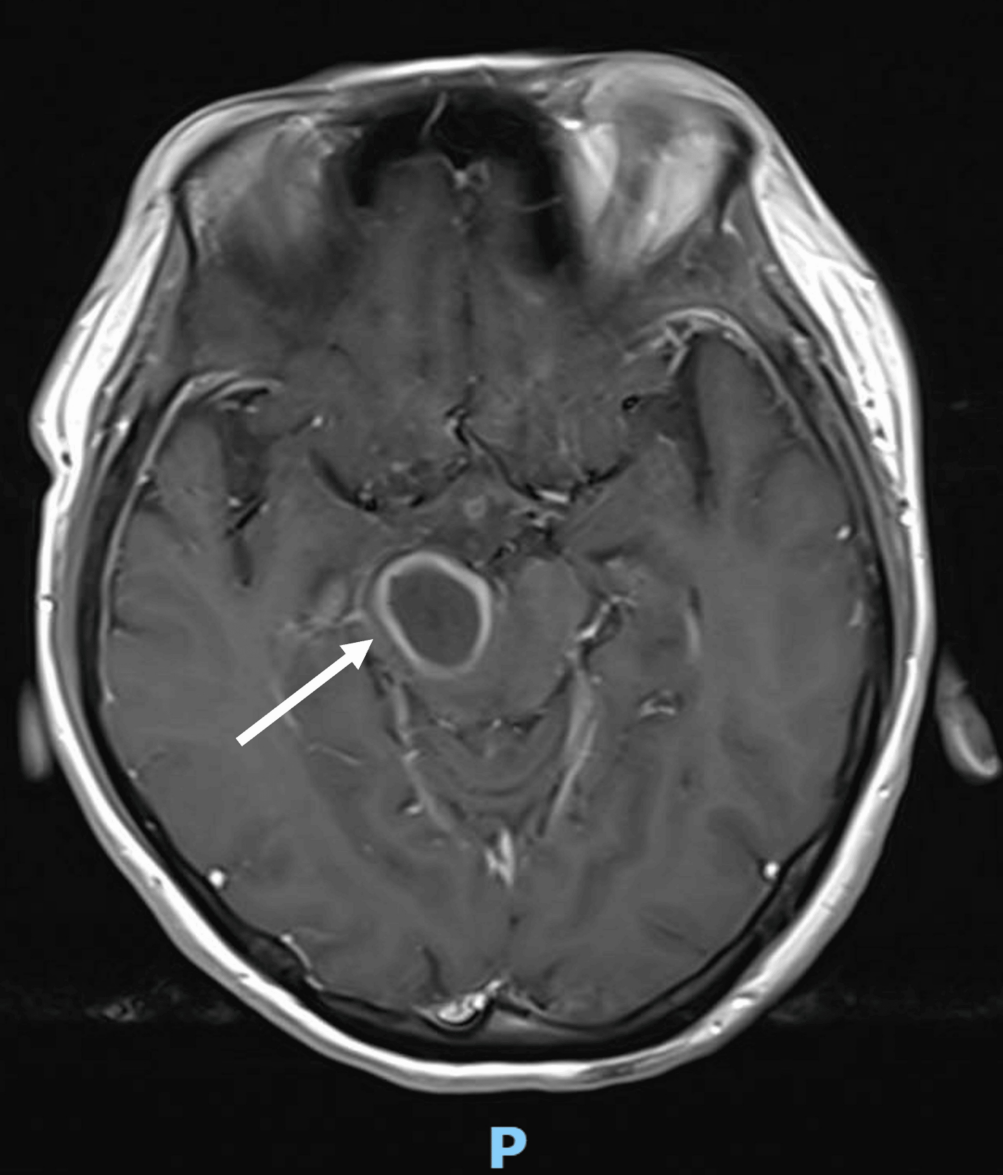
MRI brain demonstrating right midbrain/cerebral peduncle abscess (arrow)

Neurosurgery was consulted and magnetic resonance imaging (MRI) of the brain was arranged (Figure 2). It showed that the previously described CT findings corresponded to a right midbrain/cerebral peduncle ovoid lesion (2.1 x 1.8 x 2.2 cm) with marked restricted diffusion, heterogeneously hyperintense T2 signal with rim T2 hypointensity, patchy peripheral susceptibility, thick rim enhancement and local mass effect with extensive perilesional oedema extending into the brainstem, right superior cerebellar peduncle, right thalamus, and internal capsule. These features favored a cerebral abscess.

Infectious disease was consulted and intravenous co-amoxiclav was switched to intravenous ceftriaxone 2g twice daily. She was also started on intravenous ampicillin 2g every four hours for suspected neurolisteriosis and intravenous metronidazole 500mg every eight hours.

After discussion with neurosurgery, it was deemed that there was no neurosurgical contraindication for lumbar puncture. Lumbar puncture was performed due to concerns for meningitis and it revealed clear cerebrospinal fluid (CSF). Opening pressure was 21 mmHg. CSF protein was elevated at 0.68 g/L (0.15 - 0.45 g/L), white blood cell count was 4 /uL (0 - 5 /uL), glucose was 2.9 (2.2 - 3.9 mmol/L), and peripheral serum glucose was 7.0 mmol/L. CSF gram stain did not show any organisms and cultures were negative.

On the third day of admission, she reported left-sided weakness and was more lethargic. On examination, she had a new left pronator drift and Medical Research Council (MRC) motor scores of 4- in the left upper and lower extremities. The left hemiparesis in conjunction with existing right cranial nerve III palsy was consistent with Weber's syndrome due to right midbrain abscess. Neurosurgery was updated and she was started on intravenous dexamethasone. As the abscess was located in the midbrain, which contains critical neurological structures for consciousness, motor control, and cranial nerve function, it was recommended to continue intravenous antibiotics and to consider surgical aspiration of the midbrain abscess should her condition progress symptomatically or radiologically.

She was subsequently more alert and her neurological deficits remained stable. After discussion with neurosurgery, dexamethasone was oralised on the fifth day of admission with plans to discontinue it three days later.

**Figure 3:**
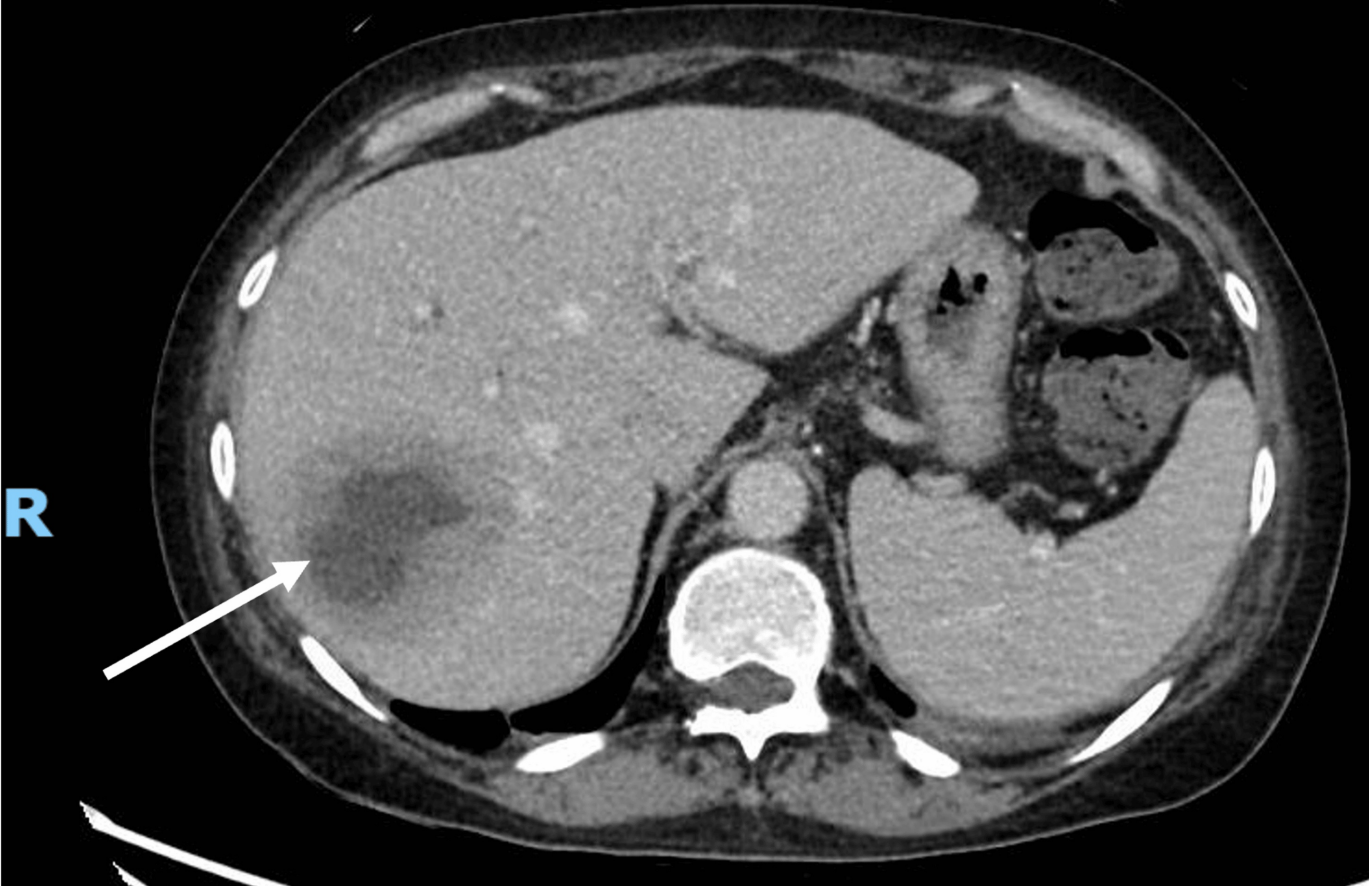
Computed tomography of the chest, abdomen and pelvis demonstrating right hepatic lobe heterogenous lesion with central hypodensity (arrow)

Computed tomography of the chest, abdomen, and pelvis, as well as a transthoracic echocardiogram, was arranged to look for the source of her midbrain abscess. The CT scans showed a 6.2 x 5.2 cm ill-defined heterogeneous lesion with central hypodensity in the right hepatic lobe, compatible with liver abscess (Figure 3).

Her transthoracic echocardiogram was unremarkable. On the sixth day of admission, she underwent image-guided drainage of the liver abscess. Purulent fluid was aspirated and aerobic culture was positive for *K. pneumoniae*. It was sensitive to amoxicillin/clavulanate, piperacillin/tazobactam, ceftriaxone, cefepime, aztreonam, ertapenem, gentamicin, ciprofloxacin, trimethoprim/sulfa, and cefazolin. It was resistant to ampicillin. Repeat blood cultures were negative. Intravenous ampicillin and metronidazole were discontinued and she was kept on ceftriaxone. She did not have evidence of endophthalmitis upon review by ophthalmology.

On day eight of admission, she developed new neurological deficits. On examination, she had worsening left hemiparesis with MRC motor scores of 3+ in her left upper and lower extremities. There were new findings of right eye near complete ophthalmoplegia. Right eye abduction was preserved. In addition, there was a limitation of vertical gaze of the left eye and convergence nystagmus on attempted up gaze. Previously noted right eye ptosis with right third nerve palsy was still present.

**Figure 4:**
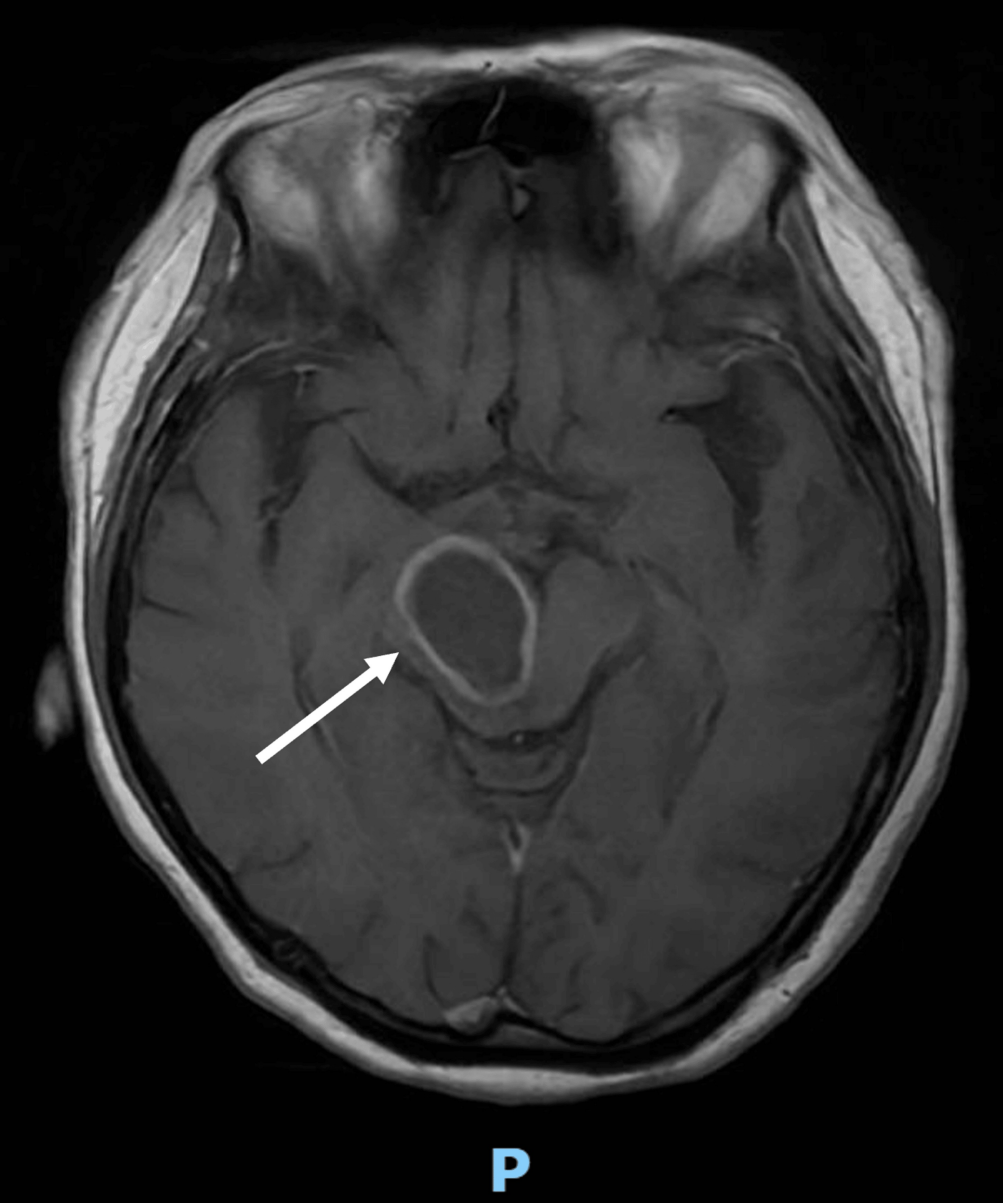
MRI Brain demonstrating interval increase in size and surrounding oedema of the known right midbrain/cerebral peduncle abscess (arrow)

She was reviewed by neurosurgery and the impression was that of worsening symptomatic right midbrain lesion with Weber's syndrome, left partial Parinaud syndrome, and right fourth cranial nerve palsy due to known midbrain abscess. MRI Brain was repeated on the same day and it showed an interval increase in size and surrounding oedema of the known right midbrain/cerebral peduncle abscess, measuring 3.0 x 2.0 x 3.1 cm (Figure 4). Intravenous dexamethasone was restarted and plans for surgical aspiration were made.

Intravenous ceftriaxone was switched to meropenem, given the enlarging midbrain abscess whilst on ceftriaxone, to cover for extended-spectrum beta-lactamase-producing *K. pneumoniae*. On Day 11 of admission, she underwent open aspiration of the right midbrain abscess. Aerobic culture of pus aspirated intraoperatively from her brain abscess was positive for *K. pneumoniae* with identical sensitivity results when compared to liver aspirate cultures. Post-operatively, her Glasgow Coma Score remained at 15. Left partial Parinaud syndrome improved, but other neurological deficits remained unchanged.

She was managed for disseminated *K. pneumoniae* infection with right midbrain abscess and right hepatic lobe abscess. Based on her intraoperative brain abscess cultures, meropenem was discontinued and switched to ceftriaxone.

Her clinical condition remained stable and she was planned for rehabilitation as she had declined functionally and required assistance for activities of daily living (ADLs). CRP and leukocytosis normalised. She reported improvement in left upper and lower extremity weakness. On examination, her right fourth nerve palsy had resolved and left eye extra-ocular movements were full without convergence nystagmus. The right third nerve palsy remained.

**Figure 5:**
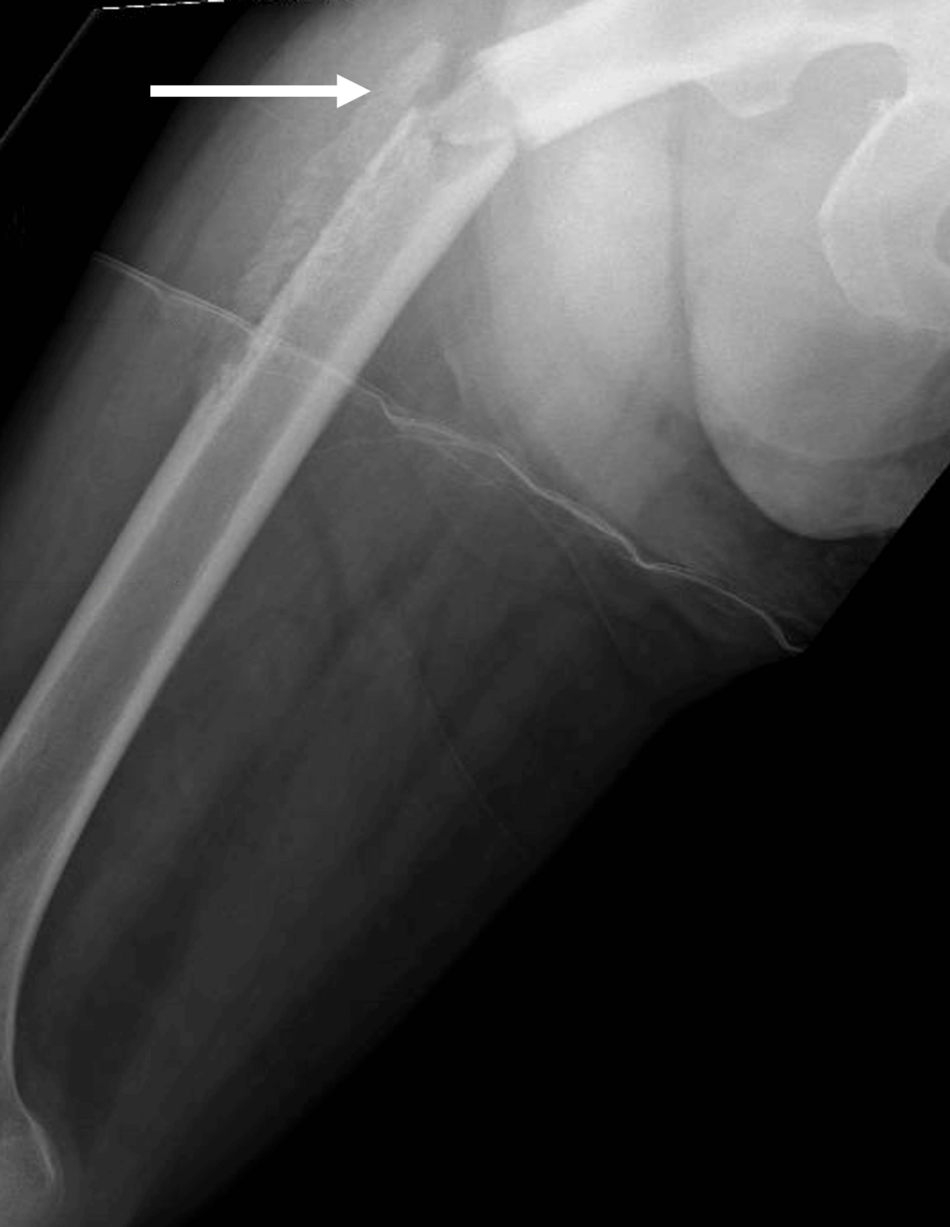
Displaced fracture (arrow) of proximal right femoral shaft on right femur radiograph

On Day 26 of admission, whilst performing exercises with physiotherapy, a crack was heard and the patient was suddenly unable to weight bear on her right lower limb. On examination, there was right thigh swelling with bruising and her right lower limb was shortened. A right femur radiograph showed a displaced fracture of the proximal right femoral shaft (Figure 5).

**Figure 6:**
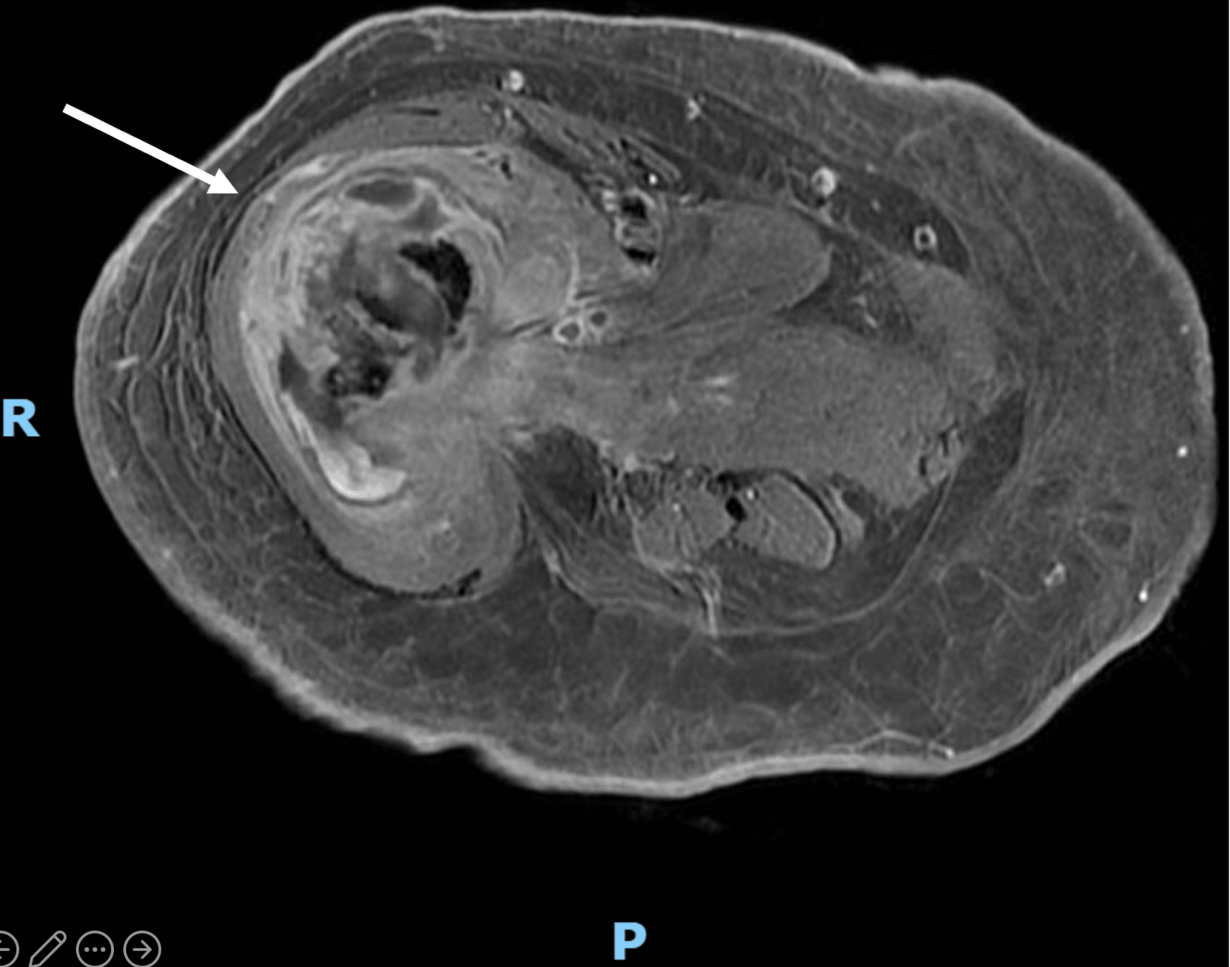
MRI of the right thigh showing multiloculated multiseptated collection (arrow) with peripheral enhancement at fracture site measuring 4.4 x 3.4 cm

Orthopedics was consulted and the impression was that of a right femur pathological fracture due to disseminated *K. pneumoniae* infection. MRI of the right thigh demonstrated right femoral pathological fracture secondary to underlying acute pyogenic osteomyelitis, given findings of intraosseous abscess associated with surrounding periosteal reaction (Figure 6). CRP was elevated at 156 mg/L (normal reference 4.9 mg/L).

Due to the presence of an intraosseous abscess and osteomyelitis despite four weeks of appropriate antibiotics, the consensus between orthopedics and infectious disease was to arrange for local debridement before planning for definitive surgery. Whilst on ceftriaxone, she developed a drug exanthem with peripheral eosinophilia, hence ceftriaxone was discontinued and switched to levofloxacin 750mg once a day.

She underwent right thigh incision and drainage and vacuum-assisted closure (VAC) application on day 45 of admission, and four days later, she underwent right thigh wound debridement and secondary closure with application of negative pressure wound therapy. Intraoperative bone and tissue cultures grew *Staphylococcus capitis*. This was treated with intravenous vancomycin and eventually oralised to trimethoprim/sulfamethoxazole. Given the clinical context, her right femur osteomyelitis and intraosseous abscess with resultant pathological fracture were attributed to disseminated *K. pneumoniae* infection. During this period, her interval CT of the abdomen and pelvis and MRI brain after seven weeks of effective antibiotic treatment showed good response.

For definitive management of her right femur fracture, options discussed included amputation or limb salvage. Subsequent interval MRI of the right thigh showed a significant reduction in the size of the intraosseous abscess with known right femoral displaced pathologic fracture due to underlying osteomyelitis. The patient eventually opted for limb salvage and was planned for outpatient orthopedic and infectious disease review. In total, she received three months of effective antibiotic treatment and there was resolution of ptosis, ophthalmoplegia, and left hemiplegia. However, she was unable to ambulate due to the pathological right femur fracture.

Table 2 provides a summary of key events that occurred during the patient's complex and prolonged hospitalisation.

**Table 2:** Summary of key events during hospitalisation

Day of admission	Key event
0	Patient presents to emergency department with diplopia and surgical right third nerve palsy.
1	Intravenous Augmentin switched to intravenous ceftriaxone 2g twice daily. Started intravenous ampicillin and metronidazole for midbrain abscess noted on MRI Brain.
2	Intravenous metronidazole stopped.
3	Patient reported left-sided weakness and was more lethargic. Found to have new left pronator drift and Medical Research Council (MRC) motor scores of 4- in the left upper and lower extremities. The left hemiparesis in conjunction with existing right cranial nerve III palsy was consistent with Weber's syndrome due to right midbrain abscess.
6	Underwent drainage of liver abscess. Aerobic culture positive for K. pneumoniae.
8	Patient developed new neurological deficits. On examination, she had worsening left hemiparesis with MRC motor scores of 3+ in her left upper and lower extremities. There were new findings of right eye near complete ophthalmoplegia. Right eye abduction was preserved. There was limitation of vertical gaze of the left eye and convergence nystagmus on attempted up gaze. Previously noted right eye ptosis with right third nerve palsy were still present. Intravenous dexamethasone restarted
9	Intravenous ceftriaxone and ampicillin stopped and switched to intravenous meropenem 2g every eight hours.
11	Underwent open aspiration of right midbrain abscess. Aerobic culture of pus aspirated intraoperatively from the brain abscess was positive for K. pneumoniae.
12	Intravenous ceftriaxone de-escalated from meropenem and dexamethasone oralised and tapered off.
17	Clinically improving after craniotomy and aspiration of right midbrain abscess. Patient reported improvement in left upper and lower extremity weakness. On examination, her right fourth nerve palsy had resolved and left eye extra-ocular movements were full without convergence nystagmus. The right third nerve palsy remained.
26	Whilst performing exercises with physiotherapy, a crack was heard and the patient was suddenly unable to weight bear on her right lower limb. Right femur radiograph showed a displaced fracture of the proximal right femoral shaft.
44	Developed drug exanthem and peripheral eosinophilia whilst on ceftriaxone. Stopped ceftriaxone and started levofloxacin 750mg once daily.
45	Underwent right thigh incision and drainage and vacuum-assisted closure application for right thigh femoral shaft osteomyelitis with intraosseous abscess and pathological fracture.
49	Underwent right thigh wound debridement and secondary closure with application of negative pressure wound therapy for right thigh femoral shaft osteomyelitis and pathological fracture.
53	Intraoperative bone and tissue cultures grew Staphylococcus capitis. Started intravenous vancomycin and continued oral levofloxacin.
60	Completed one week of intravenous vancomycin. Started oral Bactrim 960mg twice daily.
69	Resolution of right ptosis, ophthalmoplegia, and left hemiplegia.
93	Transferred to community hospital for rehabilitation.

## Discussion

Clinical manifestations of midbrain lesions

The midbrain connects the pons and cerebellum with the forebrain and can be divided into three parts: a ventral part, the tegmentum, and a dorsal part, the tectal or quadrigeminal plate [4]. The cerebral aqueduct of Sylvius is located in the midbrain. The ventral midbrain is made of the cerebral peduncles, which contain the pyramidal and corticopontine tracts. The pyramidal tracts control movements of the limbs, trunk, and cranial nerves. Within the dorsal midbrain, the nuclei of oculomotor (III) and trochlear (IV) cranial nerves are present.

Weber's syndrome refers to the constellation of ipsilateral oculomotor nerve palsy and contralateral hemiplegia or hemiparesis. It is caused by involvement of the oculomotor nucleus and cerebral peduncle in the midbrain, most commonly due to occlusion of the paramedian branches of the basilar artery or posterior cerebral artery [4]. Other causes of Weber’s syndrome include brainstem tuberculoma [5], partially thrombosed giant aneurysm of the posterior cerebral artery, primary central nervous system lymphoma [6], and intracerebral hemorrhage [7].

Parinaud syndrome (dorsal midbrain syndrome, Sylvian aqueduct syndrome) consists of supra-nuclear vertical gaze palsy secondary to lesions affecting the vertical gaze center in the dorsal midbrain [8]. It classically causes the trial of upward gaze palsy, pupillary light-near dissociation, and convergence-retraction nystagmus [4]. A retrospective observational case series of 40 cases of Parinaud syndrome found that only 65% patients had the full syndrome of vertical gaze palsy, convergence-retraction nystagmus, and light near dissociation [8].

In our case report, the patient presented with an isolated right third nerve palsy with mydriasis, suggesting a “surgical third nerve palsy,” i.e., compressive lesion on cranial nerve III. Neuroimaging findings of a right midbrain abscess correlated to her initial neurological deficits and subsequent progression, wherein she developed right fourth nerve palsy, Weber's syndrome, and left partial Parinaud syndrome. MRI with diffusion-weighted imaging has shown to be superior to CT or conventional MRI in differentiating brain abscess from other lesions, mostly brain tumors [9]. This underscores the importance of early MRI Brain with diffusion-weighted imaging in patients presenting with acute cranial nerve palsy and close monitoring for neurological deficits in patients with midbrain lesions, in particular, vertical gaze abnormalities, changes in mentation, and cerebellar and pyramidal signs. Should there be further neurological deterioration, it should prompt clinicians to arrange for urgent repeat brain imaging.

Proposed framework for the diagnosis and management of brain abscess

A brain abscess is an encapsulated area of pus within the brain parenchyma and is a life-threatening infection with significant risk of neurological deficits among survivors [9,10]. Brain abscesses usually arise from predisposing factors, such as infection with human immunodeficiency virus (HIV), history of treatment with immunosuppressive therapy, neurosurgical procedures, penetrating trauma, contiguous spread from infections (e.g., sinusitis, dental infections), or a systemic source of infection (e.g., endocarditis or bacteremia) [10]. The finding of a brain abscess on neuroimaging should prompt clinicians to evaluate for the aforementioned predisposing factors. Thus, we propose a framework for the diagnosis and management of brain abscess (Table 3).

**Table 3:** Proposed framework for diagnosis and management of brain abscess Note: This table was created by the author based on findings reported in Brouwer et al. (2014) [9,10] and Bodilsen J et al. (2024) [11] CRP: C-reactive protein, CNS: central nervous system

Steps	Assessment/Action
1. Initial neurological assessment	In patients presenting with acute or progressive cranial nerve palsies, localization of deficits should be performed on admission and regularly during admission.
	Obtain early MRI brain with diffusion-weighted imaging.
2. If imaging suggestive of brain abscess	Initiate empiric CNS-penetrating antimicrobial therapy. Antimicrobial therapy should be tailored to the patient’s demographics, history of any neurosurgical procedures or penetrating injury and geography.
	Consult infectious disease and neurosurgery.
	Explore surgical excision or drainage of brain abscess for the identification of the causative pathogen and to reduce the size of the abscess.
	Consider corticosteroids if there is increased intracranial pressure. If initiated, corticosteroid therapy should be of short duration.
	If no contraindications (e.g. brain shift on cranial imaging or coagulation disorders), and there is clinical suspicion of meningitis or abscess rupture, to arrange for lumbar puncture to obtain cultures of cerebrospinal fluid.
3. Screen for foci of infection and predisposing conditions	Perform at least two sets of blood cultures prior to administration of antimicrobial therapy and send inflammatory markers (e.g. CRP, leukocyte count).
	Look for evidence of contiguous infection (e.g. dental infection, sinusitis) and refer to appropriate specialties.
	Arrange for CT thorax, abdomen and pelvis and transthoracic echocardiogram.
	Screen for HIV and diabetes.
4. Systemic metastatic screen	Once the causative organism for brain abscess is identified, evaluation of common metastatic sites of infection should be arranged and other areas depending on patient’s symptoms and signs. For instance, if *K. pneumoniae* is identified, ophthalmology review to look for endophthalmitis should be arranged.
5. Definitive management	Patients with brain abscess will need prolonged, targeted antimicrobial therapy and require multidisciplinary care (e.g. neurosurgery, infectious disease, rehabilitation).
	For neurological sequelae, to arrange for early rehabilitation to improve patient outcomes.

Osteomyelitis

As the patient had been on ceftriaxone for several weeks prior to undergoing right thigh incision and drainage of intraosseous abscess and osteomyelitis, it could have suppressed the growth of *K. pneumoniae* in cultures taken from the bone. *Staphylococcus capitis* is a common coagulase‑negative staphylococci and a normal part of human skin flora. During orthopedic procedures, even with good sterile technique, coagulase-negative staphylococci can contaminate bone or tissue cultures. Repeated blood cultures did not demonstrate *S. capitis* growth and the clinical picture was in keeping with disseminated *K. pneumoniae* infection, with previous brain and liver abscess cultures both growing *K. pneumoniae* with identical sensitivities. Given the clinical context, her right femur osteomyelitis and intraosseous abscess with resultant pathological fracture were attributed to disseminated *K. pneumoniae* infection.

The pathological right femur fracture was an unexpected complication in our patient. Lungs, central nervous system, and eyes are the most common metastatic sites for invasive syndrome due to *K. pneumoniae* [2]. Osteomyelitis is an uncommon but documented manifestation of hypervirulent *K. pneumoniae* infection in case reports [12,13]. Due to the potential for metastatic infection, clinicians should be aware of and assess patients for such complications.

## Conclusions

This case illustrates both common (liver abscess) and uncommon complications (brain abscess, osteomyelitis) of invasive* K. pneumoniae* infection. In this case report, additional tests to confirm hypervirulent *K. pneumoniae* were not performed, and this is a limitation. We covered the clinical approach to an isolated third nerve palsy and clinical manifestations of midbrain lesions, underscoring the need for close monitoring for neurological deficits in patients with midbrain abscess. With this report, we have proposed a framework for the diagnosis and management of brain abscesses and aim to contribute to the collective knowledge in managing such cases so as to improve patient outcomes.
